# The roles of COVID‐19 pandemic exposure and telehealth in prenatal care access for rural and racial minority communities in the United States: A retrospective cohort study

**DOI:** 10.1111/jrh.70077

**Published:** 2025-09-29

**Authors:** Peiyin Hung, Jiani Yu, Adiba B. Promiti, Berry A. Campbell, Nansi S. Boghossian, Anirban Chatterjee, Bo Cai, Jihong Liu, the National COVID Cohort Collaborative Consortium

**Affiliations:** ^1^ University of South Carolina, Arnold School of Public Health, Rural Health Research Center Columbia South Carolina USA; ^2^ Department of Health Services Policy & Management Arnold School of Public Health University of South Carolina Columbia South Carolina USA; ^3^ South Carolina Smart State Center for Healthcare Quality University of South Carolina Columbia South Carolina USA; ^4^ Department of Population Health Sciences Population Health Sciences, Weill Cornell Medical College New York New York USA; ^5^ Department of Epidemiology and Biostatistics Arnold School of Public Health University of South Carolina Columbia South Carolina USA; ^6^ Division of Maternal‐Fetal Medicine University of South Carolina School of Medicine Columbia South Carolina USA

**Keywords:** pandemic, prenatal care access, race/ethnicity, rural health, telehealth

## Abstract

**Purpose:**

To examine how COVID‐19 public health emergency (PHE) exposure during pregnancy and telehealth use were associated with rural‐urban and racial/ethnic differences in prenatal care initiation timing and frequency.

**Methods:**

This retrospective cohort study of 349,682 pregnancies to birthing individuals who received both prenatal and intrapartum care at the 75 health systems in the United States contributing to the National Clinical Cohort Collaborative (N3C) from 6/1/2018 through 5/31/2022. Outcomes included prenatal care initiation timing and the number of prenatal care visits. Prenatal periods were categorized into 3 PHE exposure groups: (1) never, (2) partially, and (3) fully exposed to the PHE. The full‐exposure group was further categorized into telehealth users and those with exclusively in‐person care.

**Findings:**

The full‐exposure group with telehealth uptake had the earliest prenatal care initiation (median: 9 weeks [interquartile range: 7‐13]) and the most visits (19 visits [12‐20]). In contrast, the full‐exposure group *without* telehealth use initiated care the latest (11 weeks [8‐21]) and had the fewest visits (13 visits [6‐22]). Rural‐urban disparities persisted; however, telehealth users in both groups had earlier initiation and more visits. Racial and ethnic disparities in timeliness to initiation were most pronounced among the full‐exposure group with telehealth (Black‐White: adjusted hazard ratio [aHR]: 0.76, 95% CI, 0.70‐0.83; Hispanic‐White: aHR: 0.62, 95% CI, 0.58‐0.68), compared to the full‐exposure group with exclusively in‐person care (Black‐White: 0.95 [0.93‐0.94]; Hispanic‐White: 0.80 [0.80‐0.81]).

**Conclusions:**

Prenatal telehealth care improved early initiation but also exacerbated racial/ethnic disparities in the timeliness of prenatal care access. However, rural‐urban disparities persisted.

## INTRODUCTION

Timely access to prenatal care plays a vital role in maternal and infant health for the 4‐million women giving birth each year in the United States.^1^, ^2^ Ideally, the initial prenatal visit should occur early in gestation.^2^, ^3^ This early timing allows for accurately assessing pregnancy risks and family histories, which are essential for effective pregnancy management and monitoring.^3^, ^4^, ^5^ Early identification allows patients, families, and caregivers to make necessary adjustments and develop an optimal prenatal care plan. This plan may include recommendations on diet, weight gain, activity level, mental health care, education on pregnancy‐specific complications and their symptoms, and close monitoring of maternal and fetal health.^3^, ^6^, ^7^ Improving women's access to timely prenatal care—defined as initiating care within the first trimester—can also facilitate the identification and subsequent management of high‐risk pregnancies.^6^

Delayed prenatal care has been associated with adverse health outcomes, including low birthweight, preterm birth, stillbirth, and infant death.^8^, ^9^, ^10^ Despite this, a quarter (24.2%) of US pregnant individuals in December 2023 had their first prenatal care visit after the first trimester.^1^ The timeliness of prenatal care initiation varied substantially, with 51.9% of Native Hawaiian or Other Pacific Islander people, 63.0% of American Indian or Alaska Natives, 66.5% of Black individuals, 72.0% of Hispanic individuals, and 82.3% of non‐Hispanic white people initiating care in the first trimester.^1^ These disparities are particularly prevalent among birthing people living in rural communities, due to facility closures, lack of transportation, and delay in insurance enrollment.^11^

The COVID‐19 public health emergency (PHE) catalyzed a rapid uptake of telehealth, a modality widely recognized as a promising complement to routine prenatal care.^4^, ^12^, ^13^ Such rapid transition toward telehealth adoption provides an unprecedented opportunity to examine the disparities in its uptake. A national study found telehealth prenatal care increased from nearly none before COVID‐19 to a peak of 8.1% in November 2020.^14^ Emerging research suggests that telehealth offers greater flexibility,^4^, ^13^ which may be especially beneficial for patients who lack transportation or live in rural areas.^15^ Telehealth may also improve the set of care options available to patients, including by enabling access to subspecialists located several hours away, allowing more choices of providers for patients of color who may not feel comfortable with local options, and providing a means for more access to prenatal mental health services.^16^, ^17^, ^18^ Additionally, the option to have a telehealth visit may allow patients to get necessary care more quickly, including by assisting in recognizing pregnancy complications and mental health issues that may require immediate medical attention.^16^, ^19^, ^20^

Recognizing these opportunities, many obstetric stakeholders have begun exploring hybrid prenatal care models that combine telehealth with traditional in‐person visits.^14^ One such approach is the 4‐1‐4 prenatal care pathway, which includes 4 in‐person visits, 1 ultrasound visit, and 4 virtual visits.^21^ This model focuses on in‐person care for services that cannot be delivered remotely, while allowing for services such as anticipatory guidance and psychosocial support to be delivered virtually.^7^ Early evaluations of the 4‐1‐4 model during the COVID‐19 pandemic showed promising results, indicating that hybrid care pathways can maintain maternal and fetal outcomes while offering greater convenience and accessibility for birthing individuals.^7^, ^22^

Although clinical trials show that telehealth improves prenatal care support, the role of prenatal telehealth uptake on care timeliness and frequency remains unclear. A key question yet to be answered is whether telehealth use will reduce or widen persistent rural, racial, and ethnic disparities in timely prenatal care access. This study leverages a national dataset of birthing individuals who had prenatal care to examine the association of prenatal telehealth uptake with the timing of prenatal care, frequency of visits, as well as whether it reduces access disparities among rural and racial minority birthing people. We hypothesized that telehealth use improves timely access to prenatal care, particularly early initiation in the first trimester, and reduces visit frequency. Regarding prenatal care access disparities, we hypothesized that prenatal telehealth use would mitigate rural‐urban and racial/ethnic disparities in care timeliness and frequency compared to their prepandemic peers who received only in‐person prenatal care.

## METHODS

### Study design

This retrospective cohort study leveraged electronic health records (EHRs) from the National COVID‐19 Cohort Collaborative (N3C) to identify childbirth events for all live births and stillbirths that occurred between June 1, 2018, and May 31, 2022. N3C is a large‐scale national database specifically built to harmonize EHRs for patients who were COVID‐19–positive and COVID‐19–negative to facilitate COVID‐19 research. Additional details about the N3C data and the algorithm in this study are available elsewhere.^14^, ^23^ Study participants include individuals who received both prenatal and intrapartum care in 75 health systems across all 50 US states and the District of Columbia. Specifically, these EHR data were standardized using the Observational Medical Outcomes Partnership (OMOP) Version 5.3.1. Common Data Model. Classification coding systems, such as International Classification of Diseases, Tenth Revision, Clinical Modification (ICD‐10‐CM), Current Procedural Terminology codes, Diagnosis‐Related Group (DRG) codes, Place of Services codes, and/or Major Diagnostic Categories codes, or Standard Nomenclature of Medicine, are mapped to standard OMOP concepts based on semantic and clinical relationships, including laboratory measurements, clinical observations such as vital signs, medications, and clinical conditions. This study was deemed exempt by the University of South Carolina Institutional Review Board and the N3C Data Access Committee, with informed consent waived due to secondary data analyses.

### Study population

The study included 349,682 pregnancies nationwide. The final study cohort included 349,682 pregnancies to 349,524 individuals aged 15‐49 years who gave birth from June 1, 2018 to May 31, 2022. These birthing individuals received prenatal care services from the 75 health systems contributing to the N3C data.

### Measures

Our prenatal care access outcomes include timing of prenatal care initiation, receipt of prenatal care during the first trimester (ie, early initiation), and the total number of prenatal care visits. The time to prenatal care visit initiation is defined as the difference in days between the clinically estimated pregnancy conception date and the earliest visit date of a prenatal care service per pregnancy. This duration was then converted into weeks (ie, gestational week) to measure prenatal care initiation. The second outcome, the number of visits, was constructed by summing the total unique days of prenatal care services per person, with multiple episodes in 1 day considered as a single visit.

Key independent variables included exposure to the COVID‐19 PHE during prenatal periods, maternal urban/rural residence, and maternal race and ethnicity. We categorized exposure to the COVID‐19 PHE into 3 groups based on timing during their prenatal period: (1) never‐exposed (births during June 2018‐February 2020); (2) partially exposed (conceived before March 2020‐the onset of the COVID‐19 PHE—and gave birth during or after March 2020); and (3) fully exposed to the PHE (conceived during or after March 2020). Among those whose prenatal periods fully overlapped the PHE, we distinguished between those who received telehealth for prenatal care and those with exclusively in‐person prenatal care. All those who received telehealth (ie, telehealth users) in the sample had a combination of telehealth and in‐person visits for prenatal care; none received telehealth‐only care. Rural and urban residence was classified based on the 2023 county‐level Rural‐Urban Commuting Area (RUCA) codes.^24^ Self‐reported maternal race/ethnicity was categorized as non‐Hispanic Asian, non‐Hispanic Black, Hispanic, non‐Hispanic white, and “other” groups (eg, multiple races, American Indian/Alaska Native, and Native Hawaiian or Other Pacific Islander, or unknown).

We adopted Andersen's health care utilization model to identify relevant and observable factors for prenatal care, including age at childbirth, race, ethnicity, and residence location (rurality and region), and the following clinical factors, to assess virtual health care needs: prepregnancy body mass index classified as overweight or obese versus underweight or normal weight, smoking during pregnancy, multiple birth, pre‐existing and gestational diabetes, pre‐existing and gestational hypertension, SARS‐CoV‐2 infection during pregnancy, and depression and/or anxiety during pregnancy. These clinical characteristics were identified based on the aforementioned Common Data Model algorithm.^20^ Those with missing data on residence location were included in the analysis as a separate category.

### Study analysis

We compared maternal characteristics between birthing individuals by their prenatal PHE exposure and telehealth uptake using Chi‐square tests. Kruskal‐Wallis tests were used to compare the median distributions of gestational week of prenatal care initiation and total prenatal care visits. Since over three‐quarters of the sample did not use telehealth for prenatal care, we presented means and standard deviations (SDs) instead of medians and interquartile ranges (IQRs) for the proportion of telehealth visits per person. We then compared these distributions across maternal subgroups using analysis of variance tests.

We used Cox proportional hazard models to estimate weeks to initiation across birthing individuals with varying pandemic exposure patterns, and among the full‐exposed group, with and without telehealth care. To examine the association of pandemic exposure, telehealth use, and maternal factors with prenatal care visit frequency, we used negative binomial models, controlling for timeliness of prenatal care initiation in addition to the aforementioned covariates, except SARS‐CoV‐2 infection during pregnancy, as it was highly correlated with the COVID‐19 PHE exposure during pregnancy. In both models, we examined 2‐way interaction effects between pandemic exposure/telehealth uptake and maternal residence location, as well as race and ethnicity. Relative rate ratios (RRs) and hazard ratios (HRs) with 95% confidence intervals (CIs) were reported for each pandemic exposure group compared to the never‐exposed group, and for rural‐urban, racial, and ethnic differences in prenatal visit frequency and timeliness of initiation, respectively, within each pandemic/telehealth group. Statistical analyses were performed within the N3C Enclave using R v.4.0.2, with statistical significance set at <0.05 (2‐tailed). The analyses report followed the strengthening the reporting of observational studies in epidemiology (STROBE) guidelines for observational studies.

## RESULTS

### Study population

Among 349,682 childbirths with prenatal care in the study health systems, 162,677 (46.5%) were to non‐Hispanic white, 65,571 (18.8%) non‐Hispanic Black, 14,803 (4.2%) non‐Hispanic Asian, and 59,837 (17.1%) Hispanic (Supplementary Table A1). By pandemic exposure, 95,833 (27.4%) pregnancies had prenatal periods never‐exposed to the PHE, 92,090 (26.3%) partially exposed, and the rest were fully exposed (Table 1)—of whom 147,456 (91.2%) received only in‐person prenatal care, while 14,213 (8.8%) had at least 1 prenatal telehealth visit (Supplementary Table A2).

**TABLE 1:** Maternal characteristics by prenatal exposure to COVID‐19 pandemic and prenatal telehealth uptake.

	Prenatal pandemic exposure^a^			
	Never exposed	Partially exposed	Fully exposed	
			In‐person only	Telehealth user^b^
**All study population**, number (% of all study sample)	95,833 (27.4%)	92,090 (26.3%)	147,546 (42.2%)	14,213 (4.1%)
**Number (column %) of pregnancies**				
Urban/rural residence				
Urban	64,025 (66.8)	57,312 (62.2)	95,571 (64.8)	11,991 (84.4)
Rural	8,129 (8.5)	7,559 (8.2)	14,435 (9.8)	888 (6.2)
Unknown	23,679 (24.7)	27,219 (29.6)	37,540 (25.4)	1,334 (9.4)
**Maternal race and ethnicity**				
Hispanic/Latino	13,877 (14.5)	16,482 (17.9)	26,535 (18.0)	2,943 (20.7)
Non‐Hispanic groups:				
Asian	3,518 (3.7)	4,202 (4.6)	6,269 (4.2)	814 (5.7)
Black	19,186 (20.0)	16,083 (17.5)	27,176 (18.4)	3,126 (22.0)
White	48,095 (50.2)	42,069 (45.7)	66,580 (45.1)	5,933 (41.7)
Other races^c^	11,157 (11.6)	13,254 (14.4)	20,986 (14.2)	1,397 (9.8)
**Mother's age, years**				
15‐19	4,879 (5.1)	4,483 (4.9)	8,044 (5.5)	530 (3.7)
20‐24	16,812 (17.5)	14,676 (15.9)	25,290 (17.1)	1,862 (13.1)
25‐29	26,373 (27.5)	24,407 (26.5)	39,501 (26.8)	3,484 (24.5)
30‐34	29,543 (30.8)	28,854 (31.3)	44,600 (30.2)	4,713 (33.2)
35‐39	15,154 (15.8)	15,992 (17.4)	24,005 (16.3)	2,827 (19.9)
40‐49	3,072 (3.2)	3,678 (4.0)	6,106 (4.1)	797 (5.6)
**Prepregnancy body mass index**				
Underweight or normal weight	68,770 (71.8)	65,073 (70.7)	100,485 (68.1)	8,091 (56.9)
Overweight/obesity	27,063 (28.2)	27,017 (29.3)	47,061 (31.9)	6,122 (43.1)
**SARS‐CoV‐2 infection during pregnancy**	0 (0.0)	5,500 (6.0)	25,219 (17.1)	2,605 (18.3)
**Smoking during pregnancy**	27,063 (28.2)	9,148 (9.9)	13,513 (9.2)	1,659 (11.7)
**Pre‐existing/gestational diabetes**	10,491 (10.9)	11,217 (12.2)	17,579 (11.9)	2,686 (18.9)
**Pre‐existing/pregnancy‐induced hypertension**	14,032 (14.6)	12,878 (14.0)	23,673 (16.0)	2,784 (19.6)
**Depression and/or anxiety during pregnancy**	15,449 (16.1)	13,352 (14.5)	22,274 (15.1)	2,856 (20.1)
**Multiple birth**	4,558 (4.8)	4,323 (4.7)	7,872 (5.3)	457 (3.2)
**Gestational age at birth**				
Very preterm (≤28 weeks)	1,410 (1.5)	678 (0.7)	2,374 (1.6)	172 (1.2)
Preterm (29‐36 weeks)	9,247 (9.6)	7,789 (8.5)	16,547 (11.2)	1,460 (10.3)
Full term (≥37 weeks)	85,176 (88.9)	83,623 (90.8)	128,625 (87.2)	12,581 (88.5)
**Residence census region**				
Northeast	13,025 (13.6)	14,453 (15.7)	18,634 (12.6)	2,777 (19.5)
Midwest	28,632 (29.9)	22,924 (24.9)	38,340 (26.0)	3,605 (25.4)
South	31,464 (32.8)	29,128 (31.6)	53,825 (36.5)	6,037 (42.5)
West	4,335 (4.5)	3,328 (3.6)	7,691 (5.2)	475 (3.3)
Unknown	18,377 (19.2)	22,257 (24.2)	29,056 (19.7)	1,319 (9.3)

^a^ Pandemic exposures were categorized based on their prenatal period: (1) never exposed (prepandemic—delivered from June 2018 to February 2020); (2) partially exposed with the pandemic (conceived pregnancy before March 2020 and gave birth during or after March 2020); and (3) fully exposed within the pandemic (conceived during or after March 2020).

^b^ Telehealth users in this study had at least 1 telehealth session for prenatal care. Pregnant cohorts with telehealth prenatal care were further categorized into those using telehealth for an initial prenatal care session versus those using telehealth only for subsequent prenatal care session(s), as presented in Supplementary Table A2.

^c^ The “Other” race group includes Hawaiian or Pacific Islander, American Indian or Alaska Native, multiracial, other, or unknown. All characteristics were significantly different by pandemic exposure and telehealth uptake at *P*<.0001, according to Pearson's Chi‐square tests.

### Maternal characteristics by prenatal pandemic exposure and telehealth uptake

In the fully exposed group, Hispanic, non‐Hispanic Asian, and Black individuals were more likely to use prenatal telehealth than non‐Hispanic White individuals (Table 1). Urban residents, birthing people at more advanced ages, those with prepregnancy overweight/obesity, smokers, those with SARS‐CoV‐2 infection during pregnancy, diabetes, hypertension, and depression or anxiety during pregnancy, were more likely to use telehealth than their peers without each condition.

### Prenatal telehealth use

Roughly, one‐fourth (25.2%) of prenatal care visits by fully exposed telehealth users were through telehealth (Table 2), compared to only 0.4% of prenatal care visits for the never‐exposed group and 1.9% for the partially exposed groups (*P*<.001). The percentage of prenatal care via telehealth during the PHE was more than twice as high for Hispanic birthing people (mean [SD]: 2.7% [19.8%]), compared to non‐Hispanic white individuals (1.2% [10.3%]; *P*<.001). Urban residents had nearly triple the percentage of telehealth visits per person (2.0% [14.6%]), compared to rural residents (0.7% [6.0%]; *P*<.001).

**TABLE 2:** Distributions of prenatal care utilization by maternal characteristics.

	Week to prenatal care initiation^a^	Number of prenatal care visits^a^	% of prenatal care visits via telehealth^b^
	Median (Q1‐Q3)	Median (Q1‐Q3)	Mean (SD)
**Prenatal pandemic exposure and telehealth**			
Never exposed	10 (7‐15)	15 (8‐23)	0.4 (7.6)
Partially exposed	10 (8‐19)	13 (6‐22)	1.9 (12.7)
Fully exposed			
*Nontelehealth user*	11 (8‐21)	13 (6‐22)	0.0 (0.0)
*Telehealth user*	9 (7‐12)	19 (12‐20)	25.2 (48.3)
**Urban/rural residence**			
Urban	10 (8‐18)	15 (7‐22)	2.0 (14.6)
Rural	11 (9‐22)	15 (6‐22)	0.7 (6.0)
Unknown	11 (8‐19)	13 (6‐23)	0.9 (11.5)
**Maternal race and ethnicity**			
Hispanic/Latino	12 (9‐21)	12 (6‐20)	2.7 (19.8)
Non‐Hispanic groups:			
Asian	10 (8‐16)	14 (7‐23)	2.1 (13.2)
Black	11 (8‐19)	14 (6‐23)	1.9 (13.1)
White	10 (8‐17)	15 (7‐23)	1.2 (10.3)
Other races^b^	11 (8‐20)	14 (6‐22)	1.4 (12.4)
**Mother's age, years**			
15‐19	13 (9‐23)	13 (6‐20)	1.4 (12.1)
20‐24	12 (8‐21)	13 (6‐21)	1.3 (11.7)
25‐29	10 (8‐19)	15 (7‐22)	1.5 (12.7)
30‐34	10 (8‐17)	15 (7‐23)	1.6 (12.8)
35‐39	11 (8‐18)	13 (6‐22)	2.1 (14.4)
40‐49	11 (8‐19)	14 (7‐23)	2.7 (20.1)
**Prepregnancy body mass index**			
Underweight or healthy weight	11 (8‐20)	13 (5‐21)	1.3 (11.7)
Overweight/obesity	10 (8‐17)	17 (9‐24)	2.5 (16.4)
**Smoking during pregnancy**			
No	10 (8‐18)	14 (7‐22)	1.5 (13.1)
Yes	12 (9‐22)	11 (5‐20)	2.8 (15.4)
**Pre‐existing/gestational diabetes**			
No	11 (8‐19)	14 (6‐22)	1.4 (12.1)
Yes	11 (8‐19)	17 (9‐26)	3.2 (19.8)
**Pre‐existing and/or pregnancy‐induced hypertension**			
No	11 (8‐19)	14 (6‐22)	1.6 (13.1)
Yes	11 (8‐19)	16 (8‐25)	2.1 (14.4)
**Depression and/or anxiety during pregnancy**			
No	11 (8‐19)	14 (6‐22)	1.5 (12.5)
Yes	10 (7‐16)	17 (9‐25)	2.3 (16.9)
**Multiple birth**			
Singleton	11 (8‐19)	14 (6‐22)	1.7 (13.6)
Multiple	11 (8‐16)	16 (10‐25)	0.7 (6.3)
**Gestational age at birth**			
Very preterm (≤28 weeks)	11 (8‐20)	7 (3‐12)	1.6 (12.3)^b^
Preterm (29‐36 weeks)	11 (8‐22)	11 (5‐19)	1.6 (12.7)^b^
Full term (≥37 weeks)	11 (8‐18)	15 (7‐23)	1.6 (13.4)^b^
**SARS‐CoV‐2 infection during pregnancy**			
No	11 (8‐19)	14 (6‐22)	1.5 (12.6)
Yes	11 (8‐19)	14 (6‐23)	3.1 (18.8)
**Residence census region**			
Northeast	10 (8‐16)	13 (6‐23)	4.3 (24.2)
Midwest	10 (8‐18)	14 (6‐21)	1.5 (9.9)
South	11 (9‐20)	15 (7‐22)	1.0 (7.9)
West	11 (8‐21)	15 (6‐23)	1.7 (16.3)
Unknown	11 (8‐18)	13 (6‐24)	1.2 (12.9)

^a^ Differences in median (IQR) numbers of prenatal care visits and week to prenatal care initiation were compared using Kruskal‐Wallis tests; all were significantly different at *P*<.001.

^b^ Differences in mean (SD) percentages of prenatal care visits via telehealth were statistically significant at *P*<.001 across groups in each variable, except by gestational age at birth (*P* = .49), using analysis of variance tests.

### Prenatal care by pandemic exposure among nontelehealth users

**FIGURE 1:**
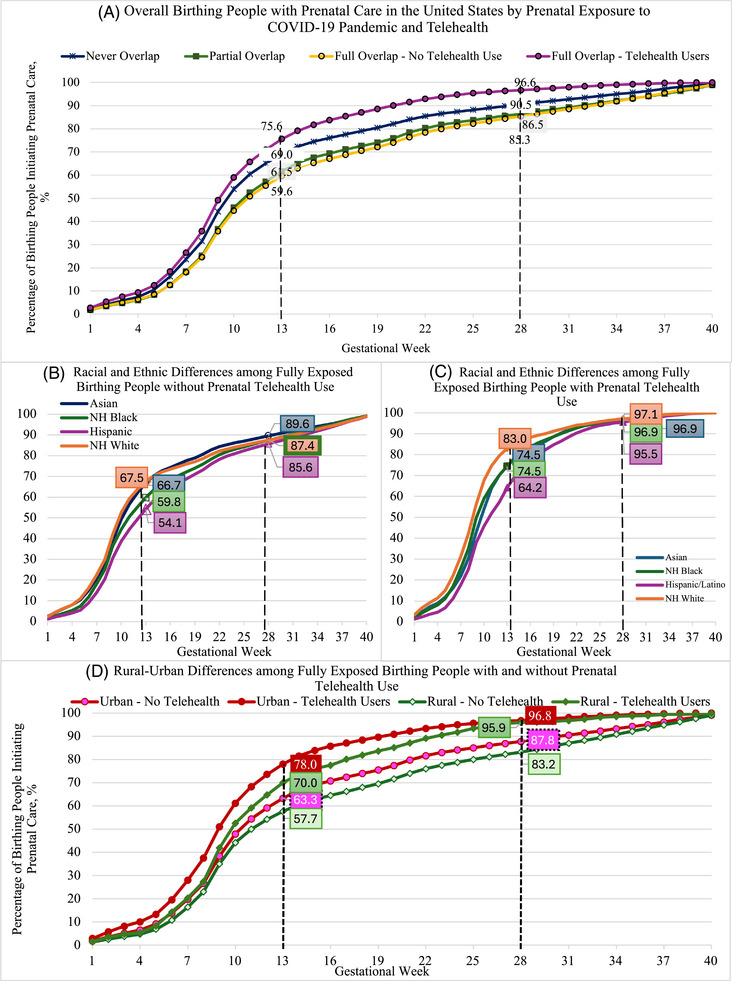
Racial/ethnic and rural disparities in weeks to prenatal care initiation among birthing people in the United States: The joint roles of pandemic exposure and telehealth use. (A) Overall birthing people with prenatal care in the United States by prenatal exposure to COVID‐19 pandemic and telehealth. (B) Racial and ethnic differences among fully exposed birthing people without prenatal telehealth use. (C) Racial and ethnic differences among fully exposed birthing people with prenatal telehealth use. (D) Rural‐urban differences among fully exposed birthing people with and without prenatal telehealth use. *Notes*: Pandemic exposures were categorized based on each birthing individual's prenatal period: (1) never exposed (birth from June 2018 to February 2020); (2) partially exposed to the pandemic (conceived pregnancy before March 2020 and birth during or after March 2020); and (3) fully exposed to the pandemic (conceived pregnancy during or after March 2020).

Overall, birthing people in the study had a median of 14 prenatal visits (IQR: 6‐22) and initiated prenatal care in the ninth gestational week (IQR: 8‐18 weeks) (Supplementary Table A1). Compared to the never‐exposed group (median [IQR]: 10 [7‐15]), those partially (10 [8‐19]) or fully exposed to the pandemic without any telehealth uptake (11 [8‐21]) had statistically significant delays in prenatal care initiation (Figure 1 and Table 2). Partially and fully exposed groups who used exclusively in‐person care also had fewer visits (median [IQR]: 13 [6‐22] for both), compared to the never‐exposed group (15 [8‐23] visits).

### Prenatal care by telehealth uptake

Telehealth users in the fully exposed period had a higher number of prenatal care visits (median [IQR]: 19 [12‐20], *P*<.0001) and earlier initiation (9 [7‐12] weeks, *P*<.0001) than any other nontelehealth user groups, regardless of pandemic exposures (Table 2). Those initiating prenatal care with telehealth had slightly fewer visits (median [IQR]: 17 [9‐24]) but similar initiation timeliness (10 [7‐15]), compared to those using telehealth for subsequent visits only (19 [12‐26] visits and 9 [7‐12] weeks, respectively; Supplementary Table A2). After covariate adjustments, individuals fully exposed to the pandemic during pregnancy without telehealth uptake had delayed initiation (adjusted hazard ratio [aHR]: 0.93, 95% CI: 0.90‐0.95; Table 3) and lower frequency of visits (adjusted rate ratio [RR], 0.94, 95% CI: 0.92‐0.96), compared to the never‐exposed group. In contrast, those fully exposed to the pandemic who used telehealth had quicker initiation (aHR, 1.45, 95% CI: 1.34‐1.56) and higher frequency of visits (RR, 1.22, 95% CI: 1.16‐1.28).

**TABLE 3:** Prenatal care initiation timeliness and visit frequency by prenatal pandemic exposure, telehealth uptake, residence location, and maternal race/ethnicity, 2018‐2022.

	Weeks to prenatal care initiation		Number of prenatal care visits	
	Hazard ratios (95% CI)^a^	*P* values	Relative rate ratios (95% CI)^b^	*P* values
**Prenatal pandemic exposure and telehealth uptake among reference group**				
Never exposed	Ref		Ref	
Partially exposed	0.94 (0.91‐0.97)	<.001	0.96 (0.94‐0.99)	.002
Fully exposed				
Nontelehealth user	0.93 (0.90‐0.95)	<.001	0.94 (0.92‐0.96)	<.001
Telehealth user	1.45 (1.34‐1.56)	<.001	1.22 (1.16‐1.28)	<.001
**Racial and ethnic disparity by prenatal pandemic exposure and telehealth use**				
Never exposed				
Non‐Hispanic Asian	1.02 (0.99‐1.06)	.18	1.05 (1.02‐1.07)	<.001
Non‐Hispanic Black	0.94 (0.92‐0.95)	<.001	1.03 (1.02‐1.04)	<.001
Hispanic/Latino	0.90 (0.89‐0.92)	<.001	0.93 (0.91‐0.94)	<.001
Non‐Hispanic White	Ref	‐	Ref	
Other races^c^	0.98 (0.96‐1.00)	.051	1.09 (1.07‐1.1)	<.001
Partially exposed				
Non‐Hispanic Asian	1.01 (1.01‐1.03)	.024	1.04 (1.02‐1.07)	<.001
Non‐Hispanic Black	0.94 (0.92‐0.94)	<.001	1.04 (1.03‐1.06)	<.001
Hispanic/Latino	0.86 (0.86‐0.87)	<.001	0.96 (0.94‐0.97)	<.001
Non‐Hispanic White	Ref	‐	Ref	
Other races^c^	0.95 (0.94‐0.96)	<.001	1.08 (1.07‐1.1)	<.001
Fully exposed—no telehealth use				
Non‐Hispanic Asian	1.02 (1.01‐1.03)	.013	1.02 (1‐1.04)	.019
Non‐Hispanic Black	0.94 (0.93‐0.94)	<.001	1.04 (1.03‐1.05)	<.001
Hispanic/Latino	0.80 (0.80‐0.81)	<.001	0.95 (0.94‐0.96)	<.001
Non‐Hispanic White	Ref	‐	Ref	
Other races^c^	0.89 (0.88‐0.89)	<.001	1.07 (1.05‐1.08)	<.001
Fully exposed—telehealth user				
Non‐Hispanic Asian	0.75 (0.66‐0.87)	<.001	0.93 (0.89‐0.96)	<.001
Non‐Hispanic Black	0.76 (0.70‐0.83)	<.001	0.97 (0.95‐1)	.023
Hispanic/Latino	0.62 (0.58‐0.68)	<.001	0.87 (0.85‐0.89)	<.001
Non‐Hispanic White	Ref	‐	Ref	
Other races^c^	0.71 (0.64‐0.78)	<.001	0.97 (0.94‐1)	.037
**Urban‐rural disparity by prenatal pandemic exposure and telehealth use**				
Never exposed				
Urban	1.26 (1.23‐1.29)	<.001	1.03 (1.01‐1.05)	<.001
Rural	Ref	‐	Ref	
Unknown	1.08 (1.05‐1.11)	<.001	0.85 (0.84‐0.87)	<.001
Partially exposed				
Urban	1.11 (1.10‐1.12)	<.001	0.98 (0.97‐1)	.08
Rural	Ref	‐	Ref	
Unknown	1.15 (1.14‐1.17)	<.001	0.83 (0.81‐0.85)	<.001
Fully exposed—no telehealth use				
Urban	1.10 (1.08‐1.09)	<.001	1.01 (1‐1.02)	.12
Rural	Ref	‐	Ref	
Unknown	1.03 (1.02‐1.03)	.002	0.87 (0.85‐0.89)	<.001
Fully exposed—telehealth user				
Urban	1.25 (1.19‐1.33)	<.001	1 (0.97‐1.04)	.82
Rural	Ref	‐	Ref	
Unknown	0.91 (0.84‐0.98)	<.001	1.21 (1.01‐1.46)	.042
**Mother's age**, **years**				
15‐19	0.87 (0.86‐0.89)	<.001	1.01 (1‐1.02)	.027
20‐24	0.92 (0.92‐0.93)	<.001	1 (0.99‐1)	.23
25‐29	Ref	‐	Ref	
30‐34	1.06 (1.05‐1.07)	<.001	1.00 (1.00‐1.01)	.35
35‐39	1.03 (1.02‐1.05)	<.001	0.94 (0.93‐0.94)	<.001
40‐49	1.00 (0.98‐1.02)	.75	1.02 (1‐1.03)	.006
**Prepregnancy overweight/obese**	1.18 (1.17‐1.19)	<.001	1.15 (1.15‐1.16)	<.001
**Pre‐existing/gestational diabetes**	0.99 (0.98‐1)	.28	1.21 (1.2‐1.22)	<.001
**Multiple birth versus singleton**	1.22 (1.2‐1.24)	<.001	1.23 (1.22‐1.24)	<.001
**Gestational age at birth**				
Very preterm (≤28 weeks)	Ref	‐	Ref	
Preterm (29‐36 weeks)	0.85 (0.82‐0.88)	<.001	1.54 (1.51‐1.58)	<.001
Full term (≥37 weeks)	0.88 (0.86‐0.91)	<.001	1.80 (1.76‐1.84)	<.001
**Pre‐existing and/or pregnancy‐induced hypertension**	0.98 (0.97‐0.99)	<.001	1.09 (1.08‐1.1)	<.001
**Depression and/or anxiety during pregnancy**	1.18 (1.17‐1.19)	<.001	1.15 (1.14‐1.16)	<.001
**Smoking during pregnancy**	0.80 (0.79‐0.8)	<.001	0.88 (0.87‐0.89)	<.001
**Early prenatal care initiation (≤13th gestational week)**	‐	‐	2.02 (2.01‐2.03)	<.001
**Region **				
Northeast	1.2 (1.19‐1.22)	<.001	1.05 (1.04‐1.06)	<.001
Midwest	1.17 (1.16‐1.18)	<.001	0.91 (0.9‐0.91)	<.001
South	Ref	‐	Ref	
West	1.00 (0.98‐1.01)	.73	1.03 (1.02‐1.05)	<.001
Unknown	1.19 (1.17‐1.21)	<.001	1.16 (1.15‐1.18)	<.001

^a^ Hazard ratios for timeliness of prenatal care initiation and *P* values were calculated from multivariable Cox proportional hazard regression models with 2‐way interactions by prenatal pandemic exposure and telehealth uptake, residence location (rural and urban), and maternal race and ethnicity.

^b^ Relative rate ratios (RR) for frequency of prenatal care visits and *P* values were calculated from a multivariable negative binomial regression.

^c^ The “Other” race group includes Hawaiian or Pacific Islander, American Indian or Alaska Native, multiracial, other, or unknown.

### Prenatal care by maternal race and ethnicity

Racial and ethnic disparities in timeliness of care (Figure 1B,C) and the frequency of visits varied across pandemic exposure groups (Table 2). Across all race and ethnicity groups, Hispanic birthing people had the fewest visits (median [IQR]: 12 [6‐20]) compared to non‐Hispanic white individuals (15 [7‐23]) and other racial minority groups. There were widening Black‐White and Hispanic‐White disparities in timeliness to initiation from never‐, partial‐, and full‐exposure groups, to the full‐exposure group using telehealth—in which group the disparities in timeliness were the most pronounced. Specifically, non‐Hispanic Black individuals had slower care initiation than their White peers with an average of 24% lower hazard of initiating care among the fully exposed telehealth users (aHR: 0.76, 95% CI: 0.70‐0.83; Table 3) and a 6% lower hazard among those without telehealth (aHR: 0.94, 95% CI: 0.93‐0.94). After adjusting for such timeliness, non‐Hispanic Black versus White individuals had higher visit frequency when not using telehealth (RR, 1.04, 95% CI: 1.03‐1.05) but lower visit frequency when using telehealth for prenatal care (RR, 0.87, 95% CI: 0.85‐0.89).

### Prenatal care by maternal residence

Rural disparities in timeliness to initiation (Figure 1D) also varied across pandemic exposure groups. Adjusted results show that urban individuals in the never‐exposed group had earlier initiation (aHR: 1.26, 95% CI: 1.23‐1.29) and higher frequency of visits than their rural counterparts care (RR, 1.03, 95% CI: 1.01‐1.05; Table 3). While rural‐urban disparities in the timeliness of initiation persisted throughout the pandemic and with telehealth uptake, such variation was not observed in visit frequency among those exposed to the pandemic—whether partially or fully during pregnancy.

## DISCUSSION

In this nationwide cohort study, only about 9% of pregnant individuals whose prenatal care periods fully overlapped with the COVID‐19 pandemic switched to prenatal telehealth care rather than relying exclusively on in‐person visits. Compared to the never‐exposed group, nontelehealth users during the fully exposed periods had later prenatal care initiation and fewer visits, whereas telehealth users initiated care earlier and had more visits. However, the benefits of earlier prenatal care initiation with telehealth use were not universal. Urban individuals consistently began care earlier than rural residents, regardless of pandemic exposure and telehealth uptake. Racial and ethnic disparities in timely prenatal care initiation were exacerbated, with the fully exposed telehealth group experiencing more pronounced disparities than the never‐exposed group—who had not benefited from rapidly adopted telehealth services.

This study contributes to the growing literature on telehealth integration in prenatal care, also aligning with previous findings on disrupted care access during COVID‐19.^1^, ^25^, ^26^ The full‐exposure group without telehealth use had, on average, 2 fewer visits than the never‐exposed group, while telehealth users had more visits, similar to patterns seen in prior observational studies involving other clinical populations, such as Medicare beneficiaries and patients undergoing mental health treatments.^27^ Our national retrospective cohort study not only provides evidence of pandemic‐related patterns of prenatal care but also disentangles the association of telehealth uptake with care frequency and timeliness during the pandemic. Our findings, showing that only 25.2% of prenatal care visits among telehealth users were conducted virtually, suggest the 4‐1‐4 hybrid model for prenatal care might not be realistic at present. Optimal hybrid care, which combines telehealth with in‐person care, has yet to be determined and requires tailoring patient care to meet individual needs through opt‐in services.^21^, ^28^

The findings that telehealth users had more prenatal visits and earlier care than nontelehealth users highlight telehealth's potential in improving care access during the pandemic. Prior research shows telehealth reduces barriers like transportation, travel burdens, and geographic limitations, allowing patients to fit appointments into their schedules more easily.^29^, ^30^ This flexibility is especially helpful for pregnant individuals with chronic or gestational comorbidities requiring frequent visits. Indeed, we found that high‐risk pregnancies were more likely to use telehealth, had more visits, and, for some, had earlier initiation of care. Yet, telehealth still accounted for a very small portion of visits, indicating the need for patient engagement in hybrid prenatal care. Telehealth, through remote monitoring and interactive virtual care, has provided enhanced psychosocial support, diagnostic tools, and therapeutic aids.^21^, ^31^ The flexibility and multifunctionality of telehealth care likely contributed to the increased visits for prenatal care, especially in cases where initial diagnostic visits could occur in‐person, followed by remote specialist care.^32^

Telehealth's uneven benefits carry implications for prenatal care equity. This study found that urban people consistently initiated care earlier than the rural group across all PHE exposure and telehealth groups. The pandemic‐era telehealth surge appeared to exacerbate racial and ethnic disparities in timely prenatal care access, especially among those with telehealth use. Contributors to these gaps are multifactorial, potentially including limited access to maternity care, technological infrastructure constraints, socioeconomic challenges, and patient skepticism/mistrust of telehealth utility in rural and minority communities.^29^, ^33^ While telehealth holds potential to improve health care access, these results highlight the importance of targeted efforts to overcome barriers to technology, reimbursement, and provider capacity to ensure telehealth improves, not worsens, health care disparities.

### Study limitations

Some limitations to consider: First, we used EHR data from 75 health systems, capturing only prenatal services provided within these settings, which may underestimate visit counts and limit generalizability to those without prenatal care and/or childbirth care in these systems. Second, due to limited telehealth uptake, users were categorized as those with at least 1 telehealth visit for prenatal care, but telehealth roles on care access might vary by modalities (eg, asynchronous, audio/phone, and video). Third, these EHR data lack some maternal factors relevant to prenatal care use, such as health insurance, income, and parity. Fourth, since N3C data were constructed for COVID‐19 research, the case‐control design might overrepresent sicker patients, limiting generalizability to the national population. Nevertheless, this large‐scale nationwide evidence fills a knowledge gap in the joint role of pandemic exposures and telehealth use during pregnancy played on access to timely prenatal care and its disparities.

### Research implications

Future research should examine telehealth effects on care quality (eg, patient satisfaction and diagnostic accuracy), efficiency (eg, costs), and clinical effectiveness (eg, pregnancy complications, maternal morbidity, and mortality), particularly among rural and minoritized individuals.^34^ Additional data on patient and provider perspectives regarding barriers and facilitators of telehealth uptake, such as digital literacy, trust in telehealth, and internet access, can help inform tailored interventions to optimize telehealth use for perinatal care in rural communities.

## CONCLUSIONS

In this cohort study of pregnant individuals with prenatal care across 75 nationwide health systems, prenatal telehealth care significantly improved the initiation timing and increased frequency of prenatal visits. This telehealth shift, however, significantly exacerbated racial/ethnic disparities in the timeliness of prenatal care initiation while rural disparities in care access persisted. These findings highlight the potential of telehealth and hybrid prenatal care as a viable avenue for addressing health care access, but patient engagement in telehealth uptake and in its integration into early prenatal care initiation is needed, particularly for rural and racial minority birthing people who have historically faced challenges in accessing timely perinatal care.

## CONFLICT OF INTEREST STATEMENT

All authors have read and approved the manuscript; all affirm that they meet authorship requirements and declare no conflicts of interest.

## Supporting information




Supporting Information

Supporting Information
