# Supplementary material for: The roles of COVID‐19 pandemic exposure and telehealth in prenatal care access for rural and racial minority communities in the United States: A retrospective cohort study
Source: J Rural Health. 2025 Sep 29;41(4):e70077. doi: 10.1111/jrh.70077 (PMC12481008; doi:10.1111/jrh.70077)
Supplement: Supplementary file 2 — Supporting Information [file JRH-41-0-s002.docx]

**Appendices**

**Appendix Methods.**

Adapting from previously published algorithm, we extracted records using the Observational Medical Outcomes Partnership (OMOP) version 5.3.1 common data model (CDM) and further limited to prenatal records from the date of estimated pregnancy conception to childbirth dates to avoid inclusions of intrapartum or postpartum records from bundled claims.

| **Variables** | Data Domain and OMOP CDM Concepts if Appropriate |
| --- | --- |
| Prenatal Records | Condition Occurrence Domain: 435887, 4063160, 72374, 72378, 72696, 72700, 73265, 73282, 75608, 76491, 76750, 76756, 76772, 77059, 78218, 78817, 78818, 79896, 80159, 80463, 81923, 192376, 192385, 193273, 193825, 193831, 195019, 196486, 196757, 196759, 197346, 200468, 313272, 315327, 315884, 432386, 432977, 433540, 433545, 434097, 434435, 435021, 435024, 435880, 435883, 435885, 436768, 437062, 437334, 437341, 437620, 437688, 437931, 437946, 439380, 439656, 440466, 80205, 134760, 135370, 194158, 195064, 198260, 199925, 200524, 258554, 258564, 260212, 260841, 313023, 313590, 319138, 374748, 433029, 436519, 438869, 439139, 606063, 606076, 606077, 3168171, 3655840, 3662256, 3663236, 4006329, 4006449, 4029320, 4034340, 4040733, 441365, 4048152, 4048166, 4048282, 4048283, 4048292, 4048460, 4048601, 4048602, 4048607, 4048610, 4048923, 4052173, 441649, 4071068, 4071069, 4071076, 4071083, 4071198, 4071717, 4071721, 4071735, 4071736, 4071737, 4071741, 4071743, 4079694, 4079851, 4079855, 4079973, 4080885, 4080888, 4082319, 4084442, 4085344, 4097959, 4105262, 4146935, 4147117, 4149586, 4162557, 4166754, 441926, 4170972, 4171091, 4171096, 4171097, 4171100, 4171102, 4171106, 4171108, 4171123, 4171691, 4172864, 4173000, 4173001, 4173002, 4173179, 4173181, 4173197, 4173198, 4173332, 442051, 4174299, 4174303, 4187201, 4237624, 4240362, 4243494, 4251487, 4258491, 4261839, 4263343, 4270073, 4278842, 4282746, 4283942, 4287783, 4293473, 4300467, 4301414, 4306199, 4306765, 4313830, 4313892, 4321550, 4322190, 4322958, 4345797, 36680586, 36712668, 36715840, 36715841, 36716757, 37017557, 37017566, 37019087, 37110521, 37119155, 42536566, 42536569, 42536745, 42537677, 42872436, 42872437, 42872438, 43021073, 44792382, 444094, 4047564, 442071, 442085, 442421, 442440, 442441, 442442, 443329, 3655881, 4025180, 4028475, ., 4028480, 4028621, 4028626, 4028629, 4028631, 4034075, 4034080, 4059763, 4059899, 4060034, 4060098, 4060240, 4060245, 4060259, 4060264, 4060559, 4060676, 4060807, 4061157, 4061347, 4061530, 4061791, 4061847, 4061971, 4062118, 4062122, 4063172, 4063175, 4063297, 4064152, 4064172, 4064175, 4064178, 4064299, 4064560, 4064722, 4064723, 4064724, 4064835, 4129015, 4129691, 4147338, 4152442, 4152443, 4152444, 4152445, 4156891, 4181975, 4310443, 35609140, 35609141, 35609142, 35609143, 37208877, 37208879, 37312479, 44793476, 44793499, 44805139, 44805140, 45763661, 45763663  Procedure Occurrence Domain: 4112701, 2211747, 2211748, 2211749, 2211750, 2211751, 2211752, 2211753, 2211754, 2211755, 2211756, 2211757, 2211758, 2211759, 2211760, 2211761, 2722250, 2211762, 2211763, 2211764, 4299735, 4327042, 4334808, 43020944, 4168545, 2110284, 44513795, 46273020, 4070024, 2110283, 2004825, 2004811, 46271899, 37016939, 4237338, 4061130, 4059697, 40492793, 4082672, 4060624, 4060626, 4231954, 4237339, 40492794, 45773218, 37396331, 42535558, 42535559, 40489798, 4197294, 3655237, 4014148, 4014149, 4014150, 4014151, 4014152, 4014433, 4014434, 4014435, 4015141, 4015142, 4015296, 4015297, 4015298, 4015299, 4015300, 4015301, 4015302, 4047845, 4060265, 4061534, 4061803, 4103871, 4141415, 4143115, 4201730, 4221720, 4250610, 4254365, 37016920, 44807850, 44808732, 44808979, 44808980, 44808981, 45763755, 4070453, 2514574, 4082749, 4233889, 4237497, 4237498, 4238529, 4254364, 4257894, 4341109, 37017962, 37109725, 37310838, 40481948, 44789441, 44810615, 44810617, 46272368, 46272582, 2108255, 40757103, 2314189  Measurement: 4014319, 4014320, 4015294, 4060266, 4061131, 4078285, 4087235, 4121342, 4191703, 4207466, 35609139, 36713462, 36713463, 36713464, 37016744, 37016760, 37310338, 37310544, 37393201, 44783612, 43020943, 4099476, 607563, 1175142, 1617152, 1617363, 3000707, 3000974, 3001059, 3001105, 3002087, 3002209, 3003169, 3003202, 3004059, 3004147, 3004222, 3004667, 3004864, 3005286, 3005749, 3005813, 3005823, 3006244, 3006855, 3006991, 3007137, 3007259, 3007898, 3007958, 3008667, 3008683, 3008867, 3009034, 3009212, 3009300, 3009518, 3009833, 3010209, 3010292, 3010506, 3012126, 3012266, 3012497, 3013324, 3014733, 3014867, 3014962, 3015257, 3015368, 3015381, 3015394, 3015873, 3016233, 3016409, 3016630, 3016758, 3017237, 3017302, 3017819, 3017938, 3018492, 3019326, 3020279, 3020352, 3021173, 3021744, 3021866, 3022447, 3022965, 3024243, 3024861, 3025305, 3025511, 3025686, 3026289, 3026348, 3026885, 3027098, 3027177, 3027334, 3027629, 3027765, 3028358, 3028596, 3028868, 3028875, 3028905, 3029141, 3029167, 3029195, 3029318, 3029440, 3029485, 3029487, 3029494, 3029645, 3029734, 3029739, 3029761, 3029767, 3029787, 3029788, 3030037, 3030065, 3030089, 3030318, 3030331, 3030342, 3030356, 3030370, 3030616, 3030627, 3030639, 3030659, 3030660, 3030668, 3030753, 3030783, 3030909, 3030922, 3030936, 3030955, 3032628, 3032802, 3033215, 3033223, 3033521, 3033849, 3034383, 3035156, 3035546, 3036322, 3036601, 3036683, 3036844, 3036934, 3037077, 3037440, 3037494, 3038036, 3038165, 3038618, 3038929, 3039154, 3040492, 3041363, 3041397, 3042795, 3042815, 3042823, 3042840, 3043110, 3043150, 3043386, 3043412, 3043437, 3043689, 3043708, 3043984, 3044815, 3044823, 3044824, 3044849, 3044851, 3044875, 3045119, 3045145, 3045167, 3045412, 3045419, 3045457, 3045689, 3045709, 3045717, 3045731, 3045991, 3046038, 3046295, 3046576, 3046602, 3046628, 3046852, 3046871, 3046886, 3046890, 3046912, 3047160, 3047186, 3047212, 3048230, 3048596, 3048886, 3049229, 3049518, 3049557, 3050129, 3050402, 3050433, 3053322, 4158633, 21492888, 21494348, 35951714, 36031183, 36031301, 36031461, 36031466, 36032422, 36203525, 36203526, 36305522, 36769326, 37019109, 37020081, 37310412, 37310413, 37310414, 37310839, 40759059, 40759089, 40762033, 40763213, 40763214, 40765088, 40765745, 43533989, 46235157, 46235160, 46236732, 46236733  Observation: 506742, 506743, 4014147, 4014321, 4015137, 4015139, 4015140, 4015295, 4047564, 4059987, 4059988, 4059989, 4060097, 4060101, 4060239, 4060241, 4060242, 4060243, 4060244, 4060246, 4060247, 4060250, 4060251, 4060258, 4060262, 4060263, 4061154, 4061156, 4061424, 4061425, 4061426, 4061428, 4061429, 4061432, 4061435, 4061437, 4061521, 4061529, 4061532, 4061786, 4061787, 4061789, 4061793, 4061802, 4062361, 4063309, 4079835, 4081292, 4083415, 4084186, 4087243, 4088025, 4137020, 4147564, 4147941, 4170305, 4173786, 4196580, 4237327, 4237344, 4237348, 4248961, 4254205, 4254206, 4257036, 4257038, 4257039, 35607944, 35609130, 35609131, 35609132, 35609133, 35609134, 35621778, 35621779, 35622068, 35622069, 35622070, 36674902, 36685904, 36685910, 36713461, 36717636, 37310337, 37310339, 37310340, 37310407, 37310408, 37310736, 40483081, 40760197, 42537958, 42689524, 43530748, 43530904, 44789715, 44793477, 44793478, 44793498, 44793526, 44793527, 44793541, 44793542, 44793543, 44793562, 44793576, 44804252, 44804550, 44808158, 44809543, 44809759, 44811395, 46285187, 4087235, 4203722, 4307024, 4330581, 601800, 762059, 1001654, 1002089, 1175602, 1175892, 1988091, 1988726, 1989262, 2101830, 2106904, 2414360, 2615489, 2615490, 2615491, 2618150, 2618151, 2618152, 2618153, 2618155, 2719397, 2719406, 2720486, 2720500, 2720904, 2721745, 3015917, 3016787, 3024174, 3030256, 3030581, 3031648, 3031834, 3032496, 3033198, 3038892, 3038905, 3039742, 3040319, 3040441, 3040643, 3174513, 3657601, 3657602, 3657603, 3657604, 3657605, 3657606, 3657607, 3657608, 3657609, 3657610, 3657611, 3657612, 3657613, 3657614, 3657619, 3657620, 3657621, 3657622, 3657623, 3657624, 4024541, 4030301, 4030911, 4045503, 4050374, 4055255, 4085346, 4114006, 4122053, 4145062, 4155796, 4170112, 4170971, 4172038, 4175499, 4189869, 4196004, 4215846, 4224773, 4264822, 4295020, 4296382, 4305726, 4310193, 4311447, 4313474, 21491261, 21491267, 21491268, 21491269, 21491270, 21491271, 21491272, 21491273, 21492587, 21492594, 21492599, 21492607, 21492611, 21492618, 21492721, 21492733, 21492738, 21492742, 21492748, 21493000, 21493785, 21493812, 21495028, 35609156, 35609175, 36031190, 36031972, 36032080, 36032327, 36203290, 36303278, 36303279, 36303318, 36303430, 36303568, 36303597, 36303600, 36303619, 36303666, 36303791, 36304061, 36304126, 36304139, 36304169, 36304222, 36304304, 36304358, 36304421, 36304609, 36304788, 36304866, 36305127, 36305164, 36305625, 36305635, 36305648, 36305690, 36305815, 36305964, 36305968, 36306076, 36306137, 36306217, 37310822, 37310823, 37310836, 37396294, 38001655, 40218315, 40218323, 40759202, 40760190, 40760192, 40760298, 40760825, 40771078, 40771079, 40771140, 40771141, 40771142, 40771144, 40771145, 40771146, 40771147, 40771148, 40771149, 40771256, 40771257, 40771258, 40771259, 40771565, 42527102, 42527158, 42527187, 42527188, 42527395, 42527523, 42528335, 42528336, 42528453, 42528454, 42528455, 42528966, 42529441, 42536210, 42579191, 42587222, 42587223, 42587224, 42587225, 42590299, 42592120, 42628127, 42628256, 42628264, 42628270, 42628295, 42628317, 42628377, 43021798, 43054890, 43533800, 43533837, 44793003, 44794767, 44794814, 44797301, 44798416, 44798468, 44799057, 44799058, 44800038, 44800947, 44804720, 44805354, 44805365, 44808043, 44808516, 44808630, 44811893, 44813328, 44814170, 44817052, 44817053, 44817089, 44817093, 44817223, 45763686, 45765514, 45765515, 45773223, 46234792, 46234841, 46235158, 46236884, 46270506, 46272536, 4219847, 4024607, 2101829, 2101831, 2108529, 2108689, 2109552  Device: 45349212, 45383356, 45764427, 45764531 |
| Telehealth | Procedure Occurrence Domain: 709752, 709752, 710056, 710057, 710058, 801014, 953288, 953289, 953290, 1314339, 1314340, 2101773, 2101774, 2314337, 2314338, 2314339, 2314340, 2514548, 2514549, 2514550, 2514551, 36716324, 37396332, 40659402, 40663506, 40663507, 44786387  Observation Domain: 603231, 608470, 608471, 608473, 608474, 608475,608476, 618899, 618900, 618901, 618902, 618903, 44812167, 764939, 953388, 1175166, 1175171, 1175199,1175262, 1175297, 1175329, 1175357, 1175366, 1175425, 1175432, 1175572, 1175587, 1175656, 1175892, 1175897,1175899, 1175962, 1175975, 1176010, 1176034, 1176047, 1176054, 1176061, 1176141, 1176162, 1176411, 1176466, 1616354,1988345, 2617473, 2617474, 2617475, 2720700, 2721636, 36032129, 36032256, 36032260, 36033671, 36203708, 36203731,36303477, 36304778, 36306033, 36659703, 36659758, 36659787, 36659852, 36659858, 36659875, 36659877, 36659894,36659898, 36659946, 36659973, 36659984, 36659985, 36659998, 36660000, 36660022, 36660047, 36660051, 36660071,36660088, 36660098, 36660179, 36660214, 36660249, 36660267, 36660278, 36660286, 36660333, 36660339, 36660418,36660442, 36660453, 36660529, 36660535, 36660621, 36660628, 36660633, 36660654, 36660655, 36660658, 36660660,36660675, 36714563, 36714564, 36717109, 36717548, 37020540, 37021046, 37395677, 37395731, 37395732, 37396521,42527874, 42528003, 42528004, 42528085, 42528086, 42528087, 42528260, 42528261, 42528262, 42528304, 42529398,42628097, 42628109, 43021425, 44783192, 44803305, 44803306, 44810185, 44812159, 45773597, 46235112, 46235113,46235114, 46285869,42628109, 40489429  Measurement: 42628422, 706416, 1620319, 1621168, 36309282, 45880527, 46237365  Visit Occurrence Domain: 5083  Device: 2618090  Meas Value: 706416, 1620319, 1621168, 46237365 |

| **Table A1. Maternal Characteristics and Prenatal Care Use among Birthing People in the Study** | |
| --- | --- |
|  | **Number (Column %) of Birthing People** |
| **All Study Population** | 349,682 |
| **Number of Prenatal Care Visits, Median (IQR)** | 14 (6-22) |
| **Number of In-Person Prenatal Visits, Median (IQR)** | 13 (5-21) |
| **Number of Prenatal Telehealth Visits among Telehealth Users, Median (Interquartile), Median (IQR)** | 2 (1-4) |
| **Week to Prenatal Care Initiation, Median (IQR)** | 9 (8-18) |
| **Urban/Rural Residence** |  |
| Urban | 228,899 (88.1) |
| Rural | 31,011 11.9) |
| Unknown | 89,772 |
| **Maternal race and ethnicity** |  |
| Non-Hispanic White | 162,677 (46.5) |
| Non-Hispanic Black | 65,571 (18.8) |
| Hispanic/Latino | 59,837 (17.1) |
| Non-Hispanic Asian | 14,803 (4.2) |
| Other Races | 46,794 (13.4) |
| **Mother's Age, Years** |  |
| 15-19 | 17,936 (5.1) |
| 20-24 | 58,640 (16.8) |
| 25-29 | 93,765 (26.8) |
| 30-34 | 107,710 (30.8) |
| 35-39 | 57,978 (16.6) |
| 40-49 | 13,653 (3.9) |
| **Pre-pregnancy Body Mass Index** |  |
| Underweight or Healthy Weight | 242,419 (69.3) |
| Overweight/Obesity | 107,263 (30.7) |
| **Smoking during Pregnancy** | 34,259 (9.8) |
| **Preexisting and/or Gestational Diabetes** | 41,973 (12.0) |
| **Preexisting and/or Pregnancy-Induced Hypertension** | 53,367 (15.3) |
| **Depression and/or Anxiety During Pregnancy** | 53,931 (15.4) |
| **Multiple Birth** | 17,210 (4.9) |
| **Gestational age at birth** |  |
| Very preterm (<28 weeks) | 4,634 (1.3) |
| Preterm (29-36 weeks) | 35,043 (10.0) |
| Full term (≥37 weeks) | 310,005 (88.7) |
| **SARS-CoV-2 Infection during Pregnancy** | 33,324 (9.5) |
| **Residence Census Region** |  |
| Northeast | 48,889 (14.0) |
| Midwest | 93,501 (26.7) |
| South | 120,454 (34.4) |
| West | 15,829 (4.5) |
| Unknown | 71,009 (20.3) |

**Table A2. Prenatal care among birthing individuals whose prenatal periods fully overlapping with the COVID-19 Pandemic by telehealth uptake**

|  | **Fully Overlapping** | | **Full Overlap-Telehealth Users** | |
| --- | --- | --- | --- | --- |
|  | **In-Person Only** | **Telehealth Users^b^** | **Telehealth User for First Visit** | **Telehealth User for Subsequent Visits Only** |
| **All Study Population, Number (% of Fully Overlapping Cohort)** | 147,546 (91.2%) | 14,213 (8.8%) | 3,182 (2.0%) | 11,031 (6.8%) |
| **Number of Prenatal Care Visits, Median (Interquartile)** | 13 (6-22) | 19 (12-20) | 17 (9-24) | 19 (12-26) |
| **% Prenatal Care via Telehealth, Mean (SD)** | 0.0 (0.0) | 25.2 (48.3) | 23.0 (37.0) | 25.9 (51.1) |
| **Week to Prenatal Care Initiation^a^** | 11 (8-21) | 9 (7-13) | 10 (7-15) | 9 (7-12) |

Notes: SD: Standard Deviation.

a. Fully overlapping group became pregnant during or after March 2020. b. Telehealth users were identified using the OMOP CDM (eMethods); this group was further classified into 1) telehealth users for the first prenatal visit and 2) telehealth users only at subsequent prenatal visits. c. Other race group includes Hawaiian or Pacific Islander, American Indian or Alaska Native, multiracial, other, or unknown. All characteristics were compared between those with and without telehealth use and between telehealth users who used telehealth for the first visit and those who used telehealth for subsequent visits with all p values <.001 according to Pearson’s chi-square tests.

**Table A3. Maternal characteristics of prenatal care patients whose prenatal periods fully overlapping with the COVID-19 Pandemic by telehealth uptake**

|  | **Fully Overlapping** | | **Full Overlap-Telehealth Users** | |
| --- | --- | --- | --- | --- |
|  | **In-Person Only** | **Telehealth Users^b^** | **Telehealth User for First Visit** | **Telehealth User for Subsequent Visits Only** |
| **Urban/Rural Residence** |  |  |  |  |
| Urban | 95,571 (64.8) | 11,991 (84.4) | 2,518 (79.1) | 9,473 (85.9) |
| Rural | 14,435 (9.8) | 888 (6.2) | 154 (4.8) | 734 (6.7) |
| Unknown | 37,540 (25.4) | 1,334 (9.4) | 510 (16.0) | 824 (7.5) |
| **Maternal race and ethnicity** |  |  |  |  |
| Hispanic/Latino | 26,535 (18.0) | 2,943 (20.7) | 769 (24.2) | 2,174 (19.7) |
| Non-Hispanic Groups: |  |  |  |  |
| Asian | 6,269 (4.2) | 814 (5.7) | 161 (5.1) | 653 (5.9) |
| Black | 27,176 (18.4) | 3,126 (22.0) | 659 (20.7) | 2,467 (22.4) |
| White | 66,580 (45.1) | 5,933 (41.7) | 1,227 (38.6) | 4,706 (42.7) |
| Other Races^c^ | 20,986 (14.2) | 1,397 (9.8) | 366 (11.5) | 1031 (9.3) |
| **Mother's Age, Years** |  |  |  |  |
| 15-19 | 8,044 (5.5) | 530 (3.7) | 108 (3.4) | 422 (3.8) |
| 20-24 | 25,290 (17.1) | 1,862 (13.1) | 457 (14.4) | 1,405 (12.7) |
| 25-29 | 39,501 (26.8) | 3,484 (24.5) | 823 (25.9) | 2,661 (24.1) |
| 30-34 | 44,600 (30.2) | 4,713 (33.2) | 1,066 (33.5) | 3,647 (33.1) |
| 35-39 | 24,005 (16.3) | 2,827 (19.9) | 580 (18.2) | 2,247 (20.4) |
| 40-49 | 6,106 (4.1) | 797 (5.6) | 148 (4.7) | 649 (5.9) |
| **Pre-pregnancy Body Mass Index** |  |  |  |  |
| Underweight or Healthy Weight | 100,485 (68.1) | 8,091 (56.9) | 1,976 (62.1) | 6,115 (55.4) |
| Overweight/Obesity | 47,061 (31.9) | 6,122 (43.1) | 1,206 (37.9) | 4,916 (44.6) |
| **SARS-CoV-2 Infection during Pregnancy** | 25,219 (17.1) | 2,605 (18.3) | 506 (15.9) | 2,099 (19.0) |
| **Smoking during Pregnancy** | 13,513 (9.2) | 1,659 (11.7) | 235 (7.4) | 1,424 (12.9) |
| **Preexisting and/or Gestational Diabetes** | 17,579 (11.9) | 2,686 (18.9) | 518 (16.3) | 2,168 (19.7) |
| **Preexisting and/or Pregnancy-Induced Hypertension** | 23,673 (16.0) | 2,784 (19.6) | 520 (16.3) | 2,264 (20.5) |
| **Depression and/or Anxiety during Pregnancy** | 22,274 (15.1) | 2,856 (20.1) | 536 (16.8) | 2,320 (21.0) |
| **Multiple Birth** | 7,872 (5.3) | 457 (3.2) | 74 (2.3) | 383 (3.5) |
| **Gestational age at birth** |  |  |  |  |
| Very preterm (<28 weeks) | 2,374 (1.6) | 172 (1.2) | 35 (1.1) | 137 (1.2) |
| Preterm (29-36 weeks) | 16,547 (11.2) | 1,460 (10.3) | 363 (11.4) | 1,097 (9.9) |
| Full term (≥37 weeks) | 128,625 (87.2) | 12,581 (88.5) | 2,784 (87.5) | 9,797 (88.8) |
| **Residence Census Region** |  |  |  |  |
| Northeast | 18,634 (12.6) | 2,777 (19.5) | 562 (17.7) | 2,215 (20.1) |
| Midwest | 38,340 (26.0) | 3,605 (25.4) | 694 (21.8) | 2,911 (26.4) |
| South | 53,825 (36.5) | 6,037 (42.5) | 1,297 (40.8) | 4,740 (43.0) |
| West | 7,691 (5.2) | 475 (3.3) | 123 (3.9) | 352 (3.2) |
| Unknown | 29,056 (19.7) | 1,319 (9.3) | 506 (15.9) | 813 (7.4) |

Notes: a. Fully overlapping group became pregnant during or after March 2020. b. Hybrid users were identified using the OMOP CDM (eMethods); this group was further classified into 1) telehealth users for the first prenatal visit and 2) telehealth users only at subsequent prenatal visits. c. Other race group includes Hawaiian or Pacific Islander, American Indian or Alaska Native, multiracial, other, or unknown. All characteristics were compared between those with and without telehealth use and between telehealth users who used telehealth for the first visit and those who used telehealth for subsequent visits with all p values <.001 according to Pearson’s chi-square tests.
